# Drug release from core-shell PVA/silk fibroin nanoparticles fabricated by one-step electrospraying

**DOI:** 10.1038/s41598-017-12351-1

**Published:** 2017-09-20

**Authors:** Yang Cao, Fengqiu Liu, Yuli Chen, Tao Yu, Deshuai Lou, Yuan Guo, Pan Li, Zhigang Wang, Haitao Ran

**Affiliations:** 1 Chongqing Key Laboratory of Ultrasound Molecular Imaging, Institute of Ultrasound Imaging, Second Affiliated Hospital, Chongqing Medical University, Chongqing, 400010 P. R. China; 2 Three Gorges Natural Medicine Engineering Research Center, School of Biological & Chemical engineering, Chongqing University of Education, Chongqing, 400067 P. R. China

## Abstract

Silk fibroin (SF), a FDA-approved natural protein, is renowned for its great biocompatibility, biodegradability, and mechanical properties. SF-based nanoparticles provide new options for drug delivery with their tunable drug loading and release properties. To take advantage of the features of carrier polymers, we present a one-step electrospraying method that combines SF, polyvinyl alcohol (PVA) and therapeutic drugs without an emulsion process. A distinct core-shell structure was obtained with the PVA core and silk shell after the system was properly set up. The model drug, doxorubicin, was encapsulated in the core with a greater than 90% drug encapsulation efficiency. Controllable drug release profiles were achieved by alternating the PVA/SF ratio. Although the initial burst release of the drug was minimized by the SF coating, a large number of drug molecules remained entrapped by the carrier polymers. To promote and trigger drug release on demand, low intensity focused ultrasound (US) was applied. The US was especially advantageous for accelerating the drug diffusion and release. The apoptotic activity of MDA-MB-231 cells incubated with drug-loaded nanoparticles was found to increase with time. In addition, we also observed PVA/SF nanoparticles that could elicit a drug release in response to pH.

## Introduction

In recent years, many attempts have been made to design and fabricate different kinds of nanoscale drug delivery systems to load therapeutic agents and release them in a controlled manner^1–3^. The design and fabrication of novel drug delivery systems is a promising and rapidly developing area. The basic requirements of a good drug delivery system include: availability of appropriate materials, the materials should be absolutely harmless to the host, and the materials must have the necessary physicochemical and biomedical properties, including the ability to degrade in biological media. A number of different carrier materials have been used for drug delivery applications; for example, synthetic and natural polymers. Some of the most commonly used synthetic polymers are composed of one or two monomers, and demonstrate controllable degradation rates. *In vivo*, their biodegradation can be predicated by such factors as molecular weight or monomer composition. Compared to the synthetic polymers, natural polymers also have good biocompatibilities. More than a dozen different kinds of nanoparticles, composed of synthetic or natural polymers, have been produced and are in early clinical development for the treatment of cancer, diabetes, and other diseases.

Silk fibroin (SF), a natural protein regenerated from the cocoons of the silkworm, *Bombyx mori*, has been used successfully in biomedicine due to its good biocompatibility, excellent mechanical properties, and tunable biodegradability^4–11^. The degradation rate of SF can be regulated by changing its molecular weight, or degree of crystallinity or crosslinking^12–14^. In recent years, an increasing number of SF-based platforms have been used for the holding and delivery of drugs^15–19^. Kaplan’s group, for example, successfully prepared doxorubicin-loaded SF nanoparticles, which acted as a stimulus-responsive anticancer nanomedicine, overcoming drug resistance mechanisms^16^. Thus, silk nanoparticles could be loaded with a therapeutic drug and show pH-dependent release *in vitro*. His group also reported a new, aqueous-based method of preparation, called polyvinyl alcohol (PVA) blends, for silk spheres with controllable sphere size and shape^20^. In these silk drug systems, the drug distribution and loading efficiency depends on their hydrophobicity and charge, which leads to different drug release profiles. Another research group produced drug-loaded magnetic SF nanoparticles with a salting-out method^21^. The regulation of SF nanoparticles and the drug entrapment was achieved by tuning the concentration of the magnetic Fe_3_O_4_ nanoparticles. By using an external magnetic field, the researchers could induce tumor targeting *in vivo* and effective chemotherapy in cases of multidrug resistance. Omenetto’s group produced SF spheres with a co-flow capillary device using PVA as the continuous phase and silk as the discrete phase^22^. The device allowed for the generation of SF spheres without further filtering. Tunable sphere diameters were achieved by adjusting the concentration of polymers, flow rate ratios, and the polymer molecular weight. The release kinetics of the silk spheres were controlled by changing the sphere size.

Differing from the above mentioned methods to produce silk drug delivery systems, coaxial electrospraying is a promising method to produce drug-loading nanoparticles in one step without an emulsion process or further purification^23–28^. The method uses electrostatic forces to eject polymer solutions in droplets and produce nanoparticles after solvent evaporation. The main advantage of this method is that it can produce drug delivery systems with polymers having different properties. Thus, novel drug delivery systems can be designed and fabricated comprised of natural and synthetic polymers. Li and his coworkers prepared cisplatin-encapsulated SF nanocarriers by electrospray without using organic solvents^29^. The *in vitro* release tests showed that the drug molecules had been incorporated into nanoparticles through a metal-polymer coordination of bond exchange and the drug could be slowly and sustainably released for more than 15 days.

To develop a controllable drug delivery system, we fabricated drug-encapsulated nanoparticles with a core-shell structure using the coaxial electrospray method. Differing from the emulsion electrospray method, coaxial electrospraying completely eliminates the emulsion step that may cause sensitive therapeutic agents to lose their bioactivity. Moreover, core-shell particles fabricated by coaxial electrospraying reduce the uncontrollable diffusion of drugs from the surface, which is one of the main disadvantages of the emulsion electrospray method for producing nanoparticles^27,30^. In the nanoparticles we produced, PVA, a FDA-approved nontoxic water soluble polymer, was used as the inner core of the nanocarriers, which were coated with natural regenerated SF layers. An anticancer drug (doxorubicin, DOX) was selected as the model drug to be incorporated in the PVA core. In addition, we investigated the influence of different ratios of PVA and SF with regards to drug release.

Despite the great efforts to design and produce novel drug delivery systems, drug efficacy in treating tumors remains limited by the relatively small amount of drug delivery and release in available systems. In current delivery systems for encapsulated drugs, only a small portion of the drug is released in the required time. The reason for the insufficient and incomplete drug release is attributed to interactions between the therapeutic drug and the carrier polymer. In addition, residual surfactant may cause an exterior hydrogel to form around the spheres, obstructing the diffusion of encapsulated drugs and restricting their complete release^27^. Therefore, to trigger the release of entrapped drugs, external stimuli can be used with the drug systems. In our study, we used external ultrasound (US) stimuli to get a controllable and continuous drug release. In addition, the release kinetics of DOX were measured at different pH values (pH 5.0, pH 6.5, and pH 7.4) to evaluate the potential of PVA/SF nanoparticles as pH-responsive, drug-delivery systems.

## Results and Discussion

### Fabrication and characterization of coaxial electrospray nanoparticles

The properties of carrier materials should be considered in terms of their composition, structure, mechanical properties, and function in producing a particular drug-delivery system. By using both natural SF and synthetic PVA polymers, coaxial electrospraying could produce nanoparticles in one step. In the set-up for the coaxial system, a dual-capillary electrospray head, comprised of two, coaxially-aligned capillaries, was used to create two separate liquid channels. The PVA solution and the SF solution were then injected in the inner and outer channels, respectively. In coaxial electrospray fabrication, the composition and diameters of the nanoparticles are greatly affected by the polymer solution characteristics and the processing parameters. To investigate the effect of the polymer concentration (PVA solution) on the PVA/SF nanoparticle size distribution, concentrations of PVA solutions were set at 0.1, 0.3, and 0.5 wt%, while the outer flow of the SF solution was set at6 wt%. The applied voltage for the coaxial electrospraying was set at 12, 16, and 20 kV. The flow rates of SF and PVA solutions were 0.2 and 0.8 ml/h, respectively. The collection distance was fixed at 90 mm.

**Figure 1:**
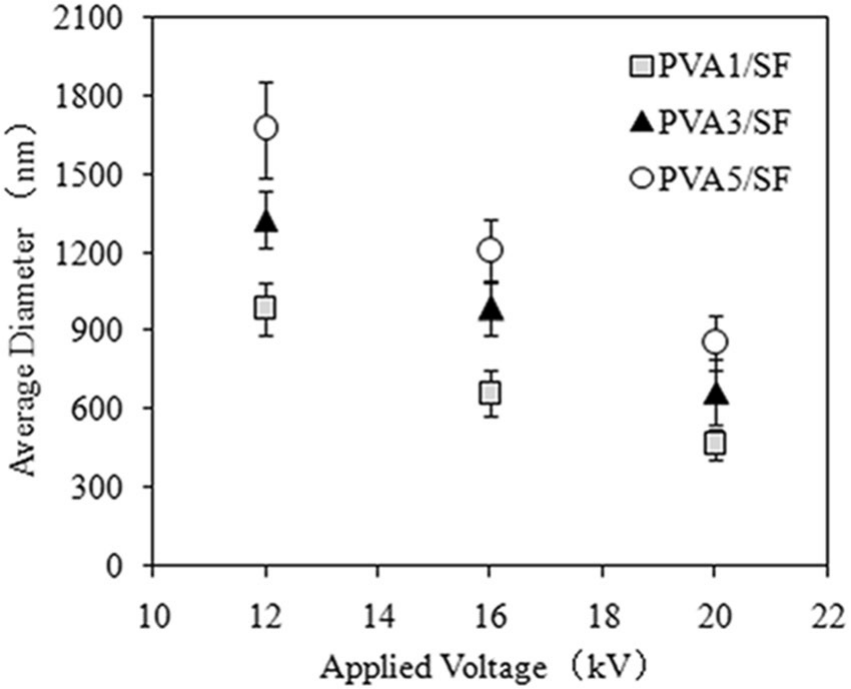
Average diameters of the PVA/SF nanoparticles.

Figure 1 shows the average diameters of the PVA/SF nanoparticles obtained by alternating the concentration of inner PVA solutions and the applied voltage of the electrospraying. At the same feeding flow rate and applied voltage (12 kV), varying the PVA concentration in the inner channel from 0.1 wt% to 0.5 wt% systematically increased the PVA/SF particle size diameters from 984 nm to 1270 nm. The same trend was also observed when the PVA/SF nanoparticles were fabricated at voltages of 16 kV and 20 kV, revealing that the size diameters of the nanoparticles were controlled by the concentration of polymers in solution. When the voltage increased from 12 kV to 20 kV, the diameters of the particles decreased correspondingly. The average diameter of PVA1/SF nanoparticles under 20 kV was distinctly smaller than that of the PVA5/SF samples, but not significantly different from the PVA3/SF samples. At an applied voltage as high as 20 kV, the polymer jet flow became unstable, and the dithering amplitude increased due to an exaggerated electric field force in the electrospray process. To obtain a stable cone-jet mode, a voltage of 16 kV was chosen, as described in the following experiments.

**Figure 2:**
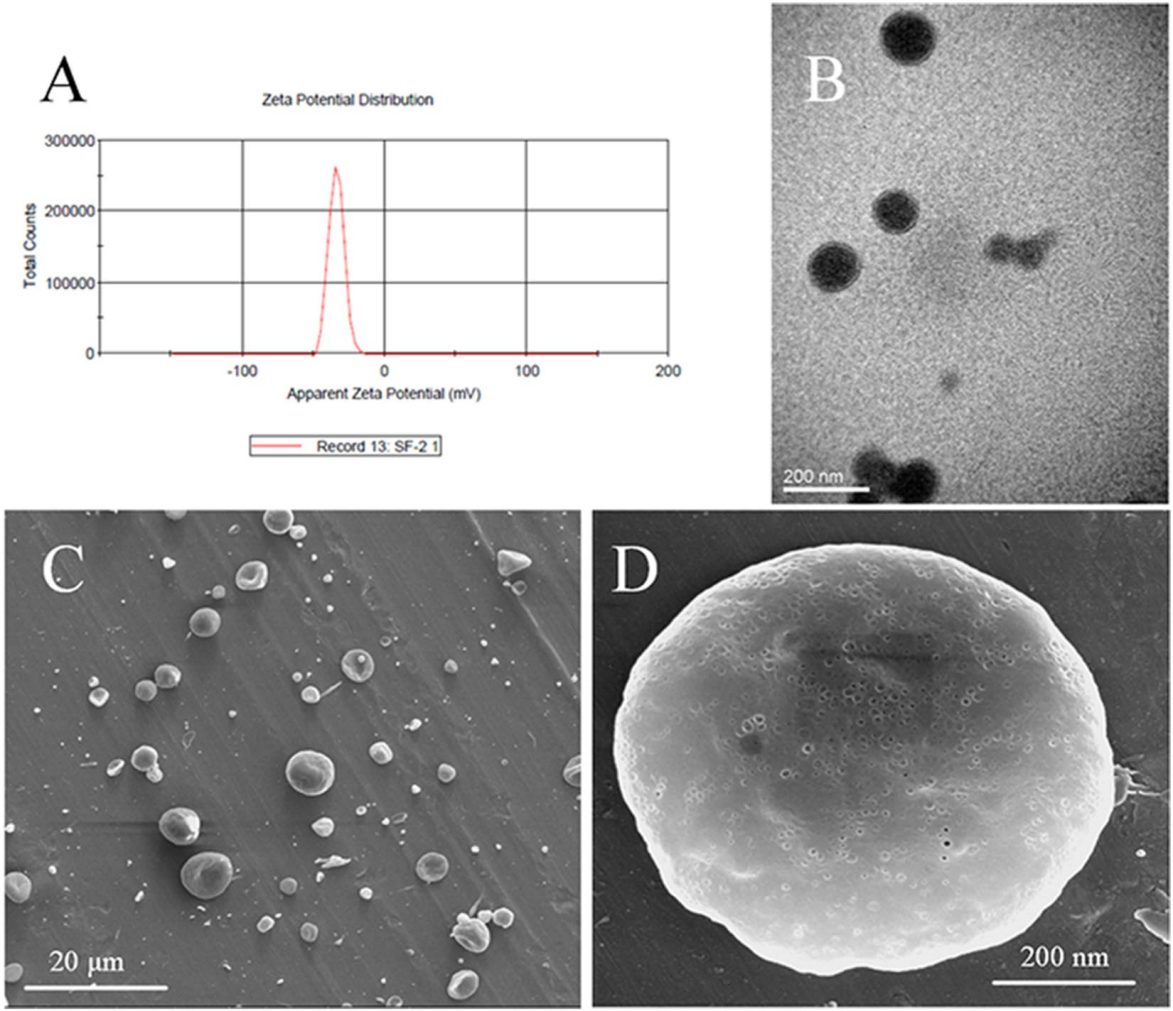
Characterization of PVA/SF nanoparticles: (**A**) surface charge distribution; (**B**) TEM image of nanoparticles; (**C**,**D**) SEM images of nanoparticles.

At a voltage of 16 kV, the morphologies of the PVA5/SF nanoparticles were characterized by scanning electron microscopy and transmission electron microscopy (Fig. 2B–D). The SEM images showed that the particles were spherical in shape and displayed a smooth surface morphology. Some mini holes were found on the surface of particles as a result of solvent volatilization during the electrospraying. The distinct core-shell structure was observed in TEM images, suggesting that the inner PVA cores had been coated by the SF. The zeta potential and surface charge of the nanoparticles has been reported to directly affect the physical stability, cellular uptake, and biodistribution of the nanoparticles *in vivo* ^31^. The surface charge of the PVA5/SF nanoparticles was −33.6 mV, which is preferred since the negative net charge may help the electrosprayed nanoparticles repel each other and prevent aggregation^32^.

### Drug release profiles *in vitro*

**Figure 3:**
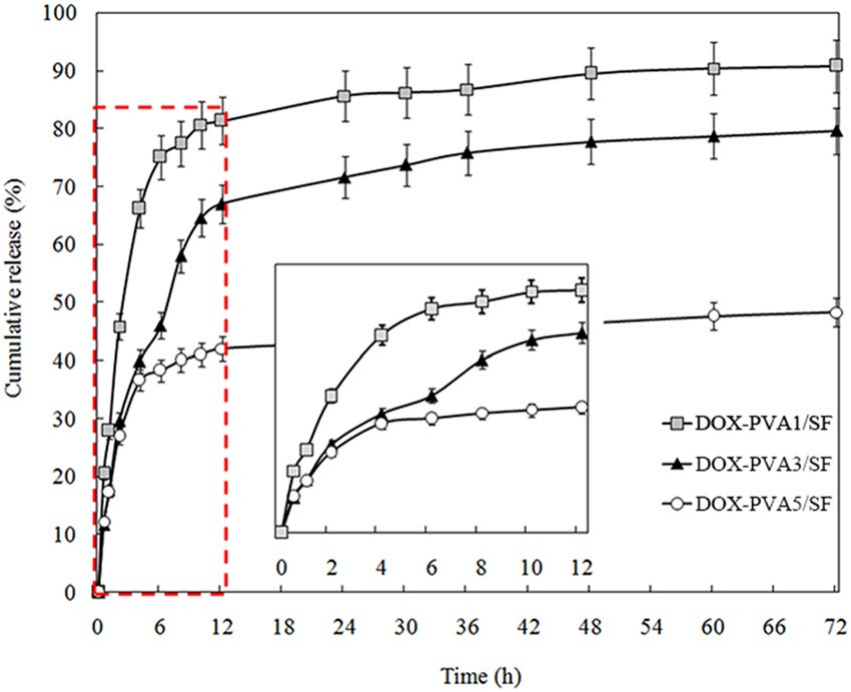
DOX release from PVA/SF nanoparticles at different PVA concentrations.

Three groups of DOX encapsulated PVA/SF nanoparticles with varying inner PVA concentrations (0.1, 0.3, and 0.5 wt%) were prepared to study their drug release profiles over 72 h (Fig. 3). Drug encapsulation efficiencies were 92.3, 95.6, and 94.7% for DOX-PVA1/SF, DOX-PVA3/SF, and DOX-PVA5/SF, respectively. The results showed that 70% of DOX was released from the DOX-PVA1/SF nanoparticles in the first 6 hour and nearly 90% was released at the end of 72 h. For the DOX-PVA3/SF and DOX-PVA5/SF groups, only 42% and 35% of the loading drugs were released, respectively. That percentage increased up to 62% for the former and 38% for the latter by 72 h. It suggested that the DOX release rate for PVA1/SF was initially higher than for the other two groups. The burst drug release was attributed to the porosity of nanoparticles that resulted from the solvent evaporation during the electrospray process. Although DOX was encapsulated in the inner region of the core-shell nanoparticles, the shell thickness of SF was too small to avoid the initial drug release, as observed from the TEM images of the nanoparticles. Furthermore, the strongly hydrophilic properties of PVA and the large surface areas of the nanoparticles could also explain the observation^33^. Due to the strong hydrophilic property of the polymer, the inner PVA was dissolved from the nanoparticles into the aqueous medium, resulting in a large number of holes in the core parts. Thus, water molecules could penetrate into the center of the nanoparticles and the loading DOX diffused and was quickly released. In addition, the polymers were likely saturated with the encapsulated drugs, leaving the remaining drug to precipitate between the carrier polymers and the outer surface, resulting in a larger initial release^34^.

As shown in Fig. 3, the DOX release percentage increased rapidly after 6 h in the DOX-PVA3/SF group. This phenomenon could be explained by the swelling of the nanoparticles. PVA, a FDA-approved ingredient in drug formulations, has been broadly used in swelling-controlled release systems. SF also has excellent swelling properties in water^29^. Large-scale swelling of PVA/SF nanoparticles has been suggested to occur when they are immersed in water, resulting in a large number of water channels in a network of drug carriers. Thus, more drug molecules would be released afterwards. Nevertheless, the accumulated release curve for the DOX-PVA5/SF samples was steady after 6 h and only 41% of the drug was released after 72 h. It indicated that a higher PVA concentration in the DOX encapsulated PVA/SF nanoparticles lead to a smaller cumulative drug release. The decreased drug release at higher mole percentages of PVA may be due to its tendency to form a denser molecular network. The interactions between encapsulated drugs and PVA molecules were also enhanced. Moreover, the exterior hydrogels of the residual PVA polymers likely obstructed the diffusion of the encapsulated DOX and restricted the completed release of drug^27^.

**Figure 4:**
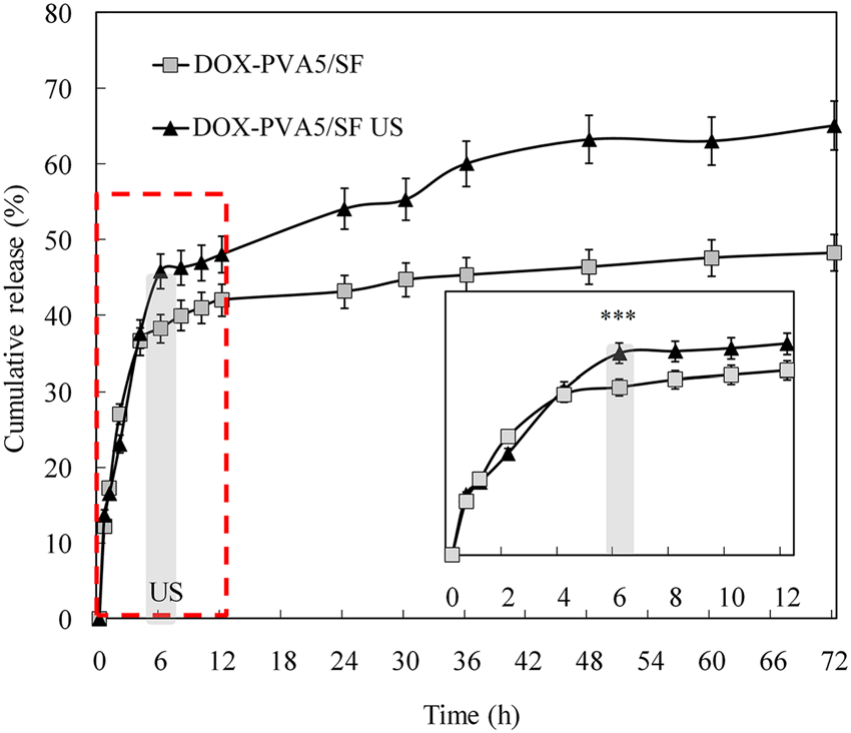
DOX release from DOX-PVA5/SF nanoparticles under ultrasound stimuli (***p < 0.01; n = 3).

In the above experiments, we hypothesized that the remaining DOX were entrapped in the PVA hydrogels after the initial drug release, leading to a slow drug release rate and the incomplete release of drug. To further investigate the release of DOX from the PVA porous gel, ultrasound (US) stimuli were used after the initial drug release period. As shown in Fig. 4, nearly 5% more DOX diffused from the PVA/SF nanoparticles after US was used at the 6th time point. More than 60% of the drug was released from the nanoparticles at 72 h, while the untreated group had released only 45% of the DOX during the same period. More than 25% of the drug was released from the nanoparticles within 72 h after US exposure. One of the universal requirements for the nanoparticles is to deliver and release their payloads at the target sites. As a kind of shock mechanical wave, US likely promotes water molecules to permeate into the polymer networks (PVA hydrogels) faster and deeper, due to the shear forces being generated by the US transducer. More drug molecules would dissolve and diffuse from the carriers. Besides influencing the drug and water molecule diffusion process, US exposure would also affect drug release after the initial release period, when the drug molecules are entrapped by the carrier polymers of the nanoparticles. The weak drug-polymer interaction may be broken by external stimuli^35^. The release kinetics of encapsulated drugs may thus be controlled for chemotherapy and these results indicate that US could cause the remaining DOX molecules trapped in surviving PVA vehicles to be quickly released and diffuse after the initial drug release.

**Figure 5:**
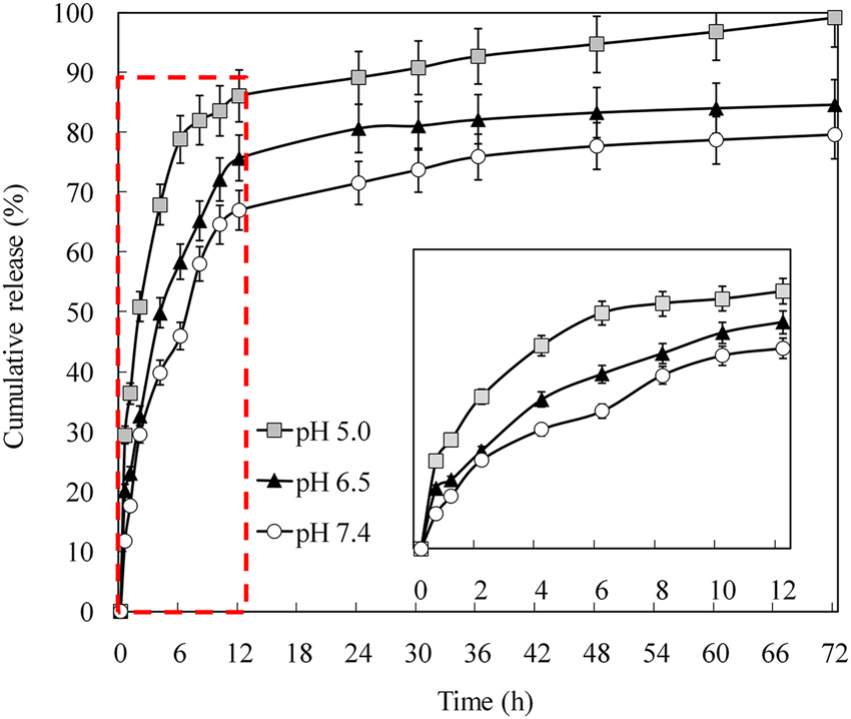
DOX release from DOX-PVA3/SF nanoparticles in different media.

Silk nanoparticles have been reported to show pH-dependent release *in vitro* ^16^. To demonstrate DOX release behaviors of PVA/SF particles under various environmental pH values, we prepared three different release buffers mimicking the microenvironment of endosomes and lysosomes (pH 5.0), the extracellular environment in tumors (pH 6.5), and blood plasma (pH 7.4)^21^. Drug release was significantly faster from the DOX-PVA3/SF nanoparticles when incubated in the medium at pH 5.0 than in either of the other two media (Fig. 5). Furthermore, 85% of the drug was released in the initial 12 h at pH 5.0. During the same time period, the initial drug release in the media at pH 7.4 and pH 6.5 were 65% and 75%, respectively. After 12 h, the drug release was constant but slow for all three conditions during the 72 h of incubation. The logD of weak bases is a critical parameter governing the drug adsorption to silk^36,37^. In core-shell nanoparticles, the silk coating had a high DOX-binding capacity at pH 7.4^36^. At low pH values, SF lost their overall acidic surface properties and had negative net charges^38^. The electrostatic interactions between DOX and SF decreased and far more drug was released^39^. It suggested that the low pH values in lysosomes could accelerate the drug release from the PVA/SF nanoparticles by changing the electrostatic interactions between the silk and DOX. Consequently, PVA/SF nanoparticles could elicit a differential drug release in response to pH changes without the need to be chemically modified.

### Cytotoxicity assay

**Figure 6:**
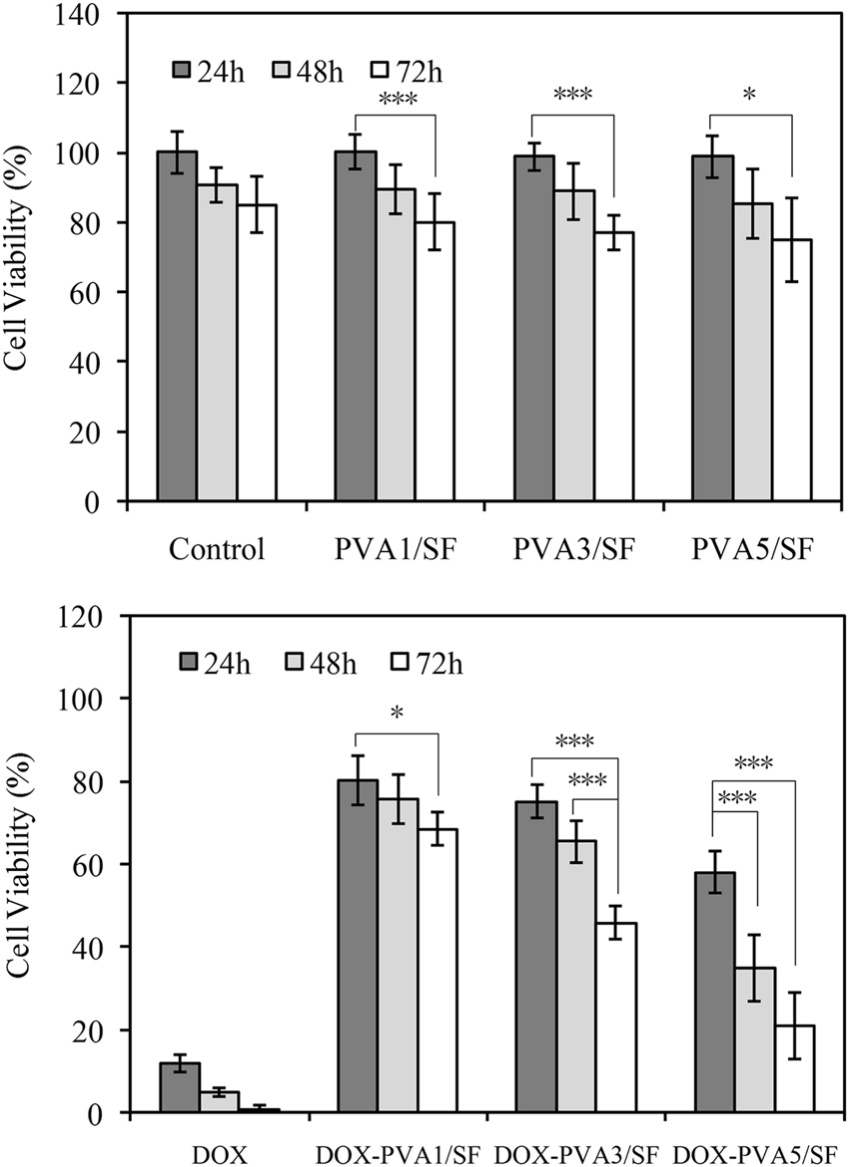
Cell cytotoxicities of PVA/SF nanoparticles: (**A**) nude particles; (**B**) DOX loading particles (*p < 0.05, ***p < 0.01; n = 3).

PVA/SF nanoparticles were incubated with MDA-MB-231 cells to investigate their cytotoxicities and chemotherapeutic effects on tumor cells. As shown in Fig. 6A, no significant difference in cell viability was observed between the PVA/SF nanoparticles (PVA1/SF, PVA3/SF, and PVA5/SF) and the control groups during the same time period, suggesting the harmlessness of the blank PVA/SF nanoparticles. When DOX was encapsulated into nanoparticles, various cell viabilities were shown for different samples (Fig. 6B). Almost 70% cell viability was found in the DOX-PVA1/SF group after 24 h incubation. The percentage increased to 73% and 78% for the DOX-PVA3/SF and DOX-PVA5/SF groups, respectively. In the DOX-PVA5/SF group, more cells (50% cell viability) survived than those of the DOX-PVA3/SF (30% cell viability) and DOX-PVA1/SF groups (20% cell viability) after 72 h of incubation. It suggested that less drug was released from the DOX-PVA5/SF nanoparticles. For extended incubation times, the cell viabilities of all of the samples decreased significantly, likely because more drugs accumulated in the medium. Compared to the samples at 48 h of incubation, cell viability was decreased by nearly 28%, 30%, and 20% for DOX-PVA1/SF, DOX-PVA3/SF, and DOX-PVA5/SF, respectively at the end of the 72 h incubation. Since a higher content of PVA can inhibit the encapsulated DOX from diffusing from the nanoparticles, a smaller amount of drug would be released from the nanoparticles, and the cell viability would be expected to be greater. Our results were in accordance with previously reported *in vitro* drug release profiles.

### Cell apoptosis

**Figure 7:**
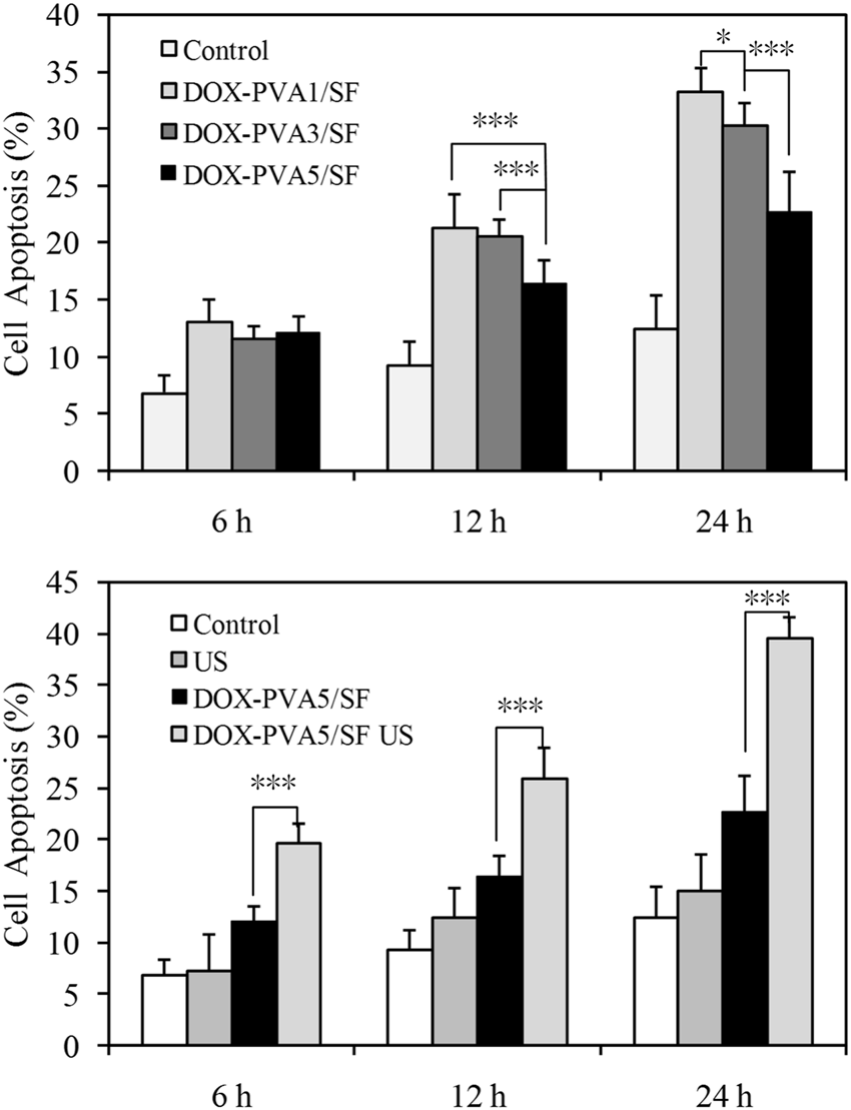
Cell apoptosis of DOX encapsulated PVA/SF nanoparticles: (**A**) nanoparticles without US exposure; (**B**) nanoparticles with US exposure (*p < 0.05, ***p < 0.01; n = 3).

Apoptotic activities of MDA-MB-231 cells were investigated using a flow cytometric analysis. As shown in Fig. 7A, after 6 h of incubation, the tumor cells in the DOX-PVA1/SF group underwent about 13% cell apoptosis. The percentage of cell apoptosis increased to 22% and 33% in 12 h and 24 h, respectively. The tumor cells in the DOX-PVA1/SF group suffered severe apoptosis, indicating that a high level of drug content had been released from the nanoparticles. In contrast, the cells in the DOX-PVA5/SF group showed less apoptosis, only 21% after 24 h of incubation. All three of the drug-encapsulated samples had similar values during the first 6 h, though the cell apoptotic activities varied with different PVA/SF ratios. In general, more cell apoptosis was seen in the groups of nanoparticles with smaller PVA concentrations since smaller amounts of the drug content was released.

US may be used as an external stimulus to control drug release behaviors. In this study, cell apoptotic activities after US exposure were evaluated (Fig. 7B). In the control group, no significant cell apoptosis was observed, indicating that the applied US did not induce cell death to a severe degree. Nevertheless, cell apoptosis increased sharply after US stimulation. For the DOX-PVA5/SF groups treated with US, the cell apoptosis rate was nearly twice that of the samples without US treatment. Since US can promote molecule movement and cell permeability, more drugs were likely released and taken up by the tumor cells (Figs S1–S3, Supporting information).

To summarize, the good compatibility of carrier polymers suggested that PVA/SF nanoparticles had no side effects after polymer degradation and erosion. By adjusting the ratio of PVA in nanoparticles and the US treatment, therapeutic drugs may be delivered and released to target sites with high efficiency. The PVA/SF nanoparticles also showed pH-dependent drug release, which could be used for stimulus-responsive nanomedicines. The PVA/SF nanoparticles could serve in the controllable release of drugs for tumor chemotherapy.

## Conclusion

SF and PVA are FDA-approved biomaterials used for drug delivery and biomedical applications. To take advantages of the properties of these polymers, we fabricated drug-loaded PVA/SF nanoparticles with distinct core-shell structures using a coaxial electrospray technology. The encapsulated drug release profiles could be altered by changing the PVA ratio in the nanoparticles. DOX was slowly and steadily released after the initial burst release over the 72 h of incubation due to barriers of the carrier polymers. Furthermore, drug release can be accelerated on demand by US treatment. In addition, the PVA/SF nanoparticles also showed pH-dependent release *in vitro* and more drugs could be released in acidic media. The assay for cell apoptosis showed that the sustained release of DOX led to high cytotoxicity for tumor cells in a time-dependent manner. US stimulation, causing a controllable release of encapsulated drug from the PVA/SF nanoparticles may allow cytotoxic drugs to accumulate at higher levels in tumors with fewer side effects.

## Materials and Methods

### Materials

*Bombyx mori* (silkworm) cocoons were obtained from Sericultural Research Institute, Chinese Academy of Agricultural Sciences. PVA (MW = 25 000) was purchased from Sigma-Aldrich, US, and doxorubicin hydrochloride (DOX) was purchased from J&K China Chemical Ltd. (Beijing, China). LiBr was obtained from Tianjin Guangfu, China. All other reagents were of analytical grade and purchased from Sinopharm Chemical Reagent, China.

### Preparation of the regenerated silk fibroin solutions

Cocoons of silk worms were boiled twice in 0.02 M Na_2_CO_3_ solution for 0.5 h and then rinsed with distilled water to remove sericin from the surface of the silk fibers. The degummed SF was dissolved in 9.3 M LiBr at 60 °C for 4–5 h. The solution was dialyzed (MW = 3.5–5 kDa) against distilled water for 3 days to remove the ions and other impurities. Finally, the SF solution was collected, filtered, and stored at 4 °C.

### PVA/Silk nanoparticle fabrication by coaxial electrospraying

Electrospraying of polymer solutions was done by injection through two flow channels with separate syringe pumps (TJ-3A, Longer, China). PVA and SF were used for the inner and outer polymers, respectively. Concentrations of the PVA ethanol solution were set at 0.1, 0.3, and 0.5 wt%. SF was dissolved in the hexafluoroisopropanol (HFIP) solution at 6 wt%. The nanoparticles were labeled as PVA1/SF, PVA3/SF, and PVA5/SF. DOX, used as the model drug, was dissolved in PVA solutions at 1% w/w. The flow rates of the inner and outer liquids were set at 0.2 and 0.8 ml/h, respectively. A coaxial nozzle was used and high electrostatic voltages were applied to the nozzle from 12 kV to 20 kV by means of a crocodile clip. The distance between the needle tip and the collector was 90 mm. The resulting droplets were continuously collected by an electrically-grounded aluminum dish with 10 ml of water, and then freeze-dried for 24 h before being tested.

### Characterization of PVA/SF nanoparticles

Average size distribution and zeta potential of the PVA/SF nanoparticles were measured by dynamic light scattering (DLS) methods using a Zetasizer Nano ZS 90 instrument (Malvern Instruments Ltd., Malvern, UK). The morphology of the particles was characterized by field emission scanning electron microscopy (FE-SEM, Nova 400, FEI, USA) and transmission electron microscope (TEM, FEI Tecnai 10, Philips Electron Optics, Holland).

### Drug release profiles *in vitro*

The DOX-loaded PVA/SF nanoparticles were collected and incubated in a conical flask with 20 ml of PBS, shaken at 37 °C, 120 rpm for 72 h. In this study, an US stimulus was used to investigate the drug release kinetics under US treatment. The transducer of the low-intensity focused ultrasound (1 MHz, acoustic intensity: 1.2 W/cm^2^, duty cycle: 50%, Chongqing Medical University, China) applied the stimulus. The acoustic power and stimulation time was set at 0.6 w and 30 s, respectively. At the designated time, 2 ml of the release medium was taken and replaced by the same amount of fresh buffer. DOX concentration was determined at 483 nm by UV spectrometry (Lambda 900, PerkinElmer, USA). The accumulated release percentage of the drug and the entrapment efficiency were evaluated according to Peltonen *et al*.^34^.

### Cell viability assay

Cell cytotoxicity was determined using the MTT assay. Human breast cancer cells (MDA-MB-231) were used to study the cytotoxicity of the PVA/SF nanoparticles. MDA-MB-231 cells were routinely cultured in high glucose DMEM with 10% fetal bovine serum (FBS) and seeded into a 96-well plate at a density of 2 × 10^4^ cells per well at 37 °C, 5% CO_2_. After 24 h of incubation, the fresh culture medium containing the PVA/SF nanoparticle samples were added into the wells. At the end of the incubation (24 h, 48 h, and 72 h), the culture medium was replaced with 20 μL MTT solution (5 mg/ml, w/v) and 180 μL serum-free medium. The plates were incubated for 4 h at 37 °C, 5% CO_2_. The medium was then withdrawn, and 150 μL DMSO was added to dissolve the formazan crystals. Absorbance was recorded at 490 nm by the microplate spectrophotometer system. Percent viability was expressed as absorbance in the presence of test compound as a percentage of absorbance of the vehicle control.

### Cell apoptosis analysis by flow cytometry

MDA-MB-231 cells were seeded in a 24-well plate at a density of 2 × 10^5^ cells per well at 37 °C, 5% CO_2_. After 24 h of incubation, fresh culture medium containing the PVA/SF DOX-loaded nanoparticle samples was added into the wells. At the end of the incubation times (24 h, 48 h, and 72 h), MDA-MB-231 cells were collected and washed with PBS. Cell apoptosis analysis was carried out with a two-color flow cytometric analysis, where cells were stained with reagents from an Apoptosis Assay Kit (Bestbio Co. Ltd., China) and analyzed with a flow cytometer (FACS CantoTM II system; BD Biosciences, USA).

### *In vivo* measurements

Four-week-old BALB/c female nu/nu mice were supplied by the Laboratory Animal Center in Chongqing Medical University. All experimental protocols were approved by the Institutional Animal Care and Use Committee of Chongqing Medical University. All the experimental operations of animals were carried out in accordance with the protocol approved by the Institutional Animal Care and Use Committee of Chongqing Medical University. All animals were treated according to the guidelines of the Care and Use of Laboratory Animals.

BALB/c nude mice xenogratted with MDA-MB-231 cells were randomly divided into three groups. The control group was treated with saline. The ultrasound group was sonicated by ultrasound. In the treatment group, samples (0.2 mL) were administrated via the tail vein. After six hours, the tumor sites were sonicated by LIFU.

The tumor volume was monitored by caliper measurement and calculated by the following equation: Volume = 0.5 × *L* × *W* ^2^. L and W are the length and the width of the tumors, respectively. The survival study was determined based on death date from the date of the first injection of each group. The accumulated amounts of DOX in various organs were investigated. After treatments, mice were scarified and the main organs (tumor, kidney, spleen, lung, liver, heart) were excised, weighed and extracted with two volumes of acetonitrile/methanol (1:1, v/v). The obtained suspensions were centrifuged and filtered through a 0.22 μm pore cellulose acetate membrane. Drug concentrations in solutions were determined using a UV spectrophotometer.

### Statistical analysis

All experiments were performed in triplicate, and the results are expressed as mean ± standard deviations. Statistical analyses of the experimental data from the different groups were performed with a one-way ANOVA. A value of p < 0.05 was considered significant, and p < 0.01 was considered highly significant.

## Electronic supplementary material

Supporting information
