# Supplementary material for: Drug release from core-shell PVA/silk fibroin nanoparticles fabricated by one-step electrospraying
Source: Sci Rep. 2017 Sep 20;7:11913. doi: 10.1038/s41598-017-12351-1 (PMC5607240; doi:10.1038/s41598-017-12351-1)
Supplement: Supplementary file 1 — Supporting information [file 41598_2017_12351_MOESM1_ESM.pdf]

Supporting information:

Drug release from core-shell PVA/silk fibroin nanoparticles fabricated by one-step electrospraying

Yang Cao,[1] Fengqiu Liu,[1] Yuli Chen,[1] Tao Yu,[1] Deshuai Lou,[2] Yuan Guo,[1] Pan Li,[1] Zhigang Wang,[1] and Haitao Ran[1]*

[1]Chongqing Key Laboratory of Ultrasound Molecular Imaging, Institute of Ultrasound Imaging, Second Affiliated Hospital, Chongqing Medical University, Chongqing, 400010, P. R. China.

[2]Three Gorges Natural Medicine Engineering Research Center, School of Biological & Chemical engineering, Chongqing University of Education, Chongqing 400067, P. R. China

*Corresponding author: Haitao Ran. Email: rht66@163.com

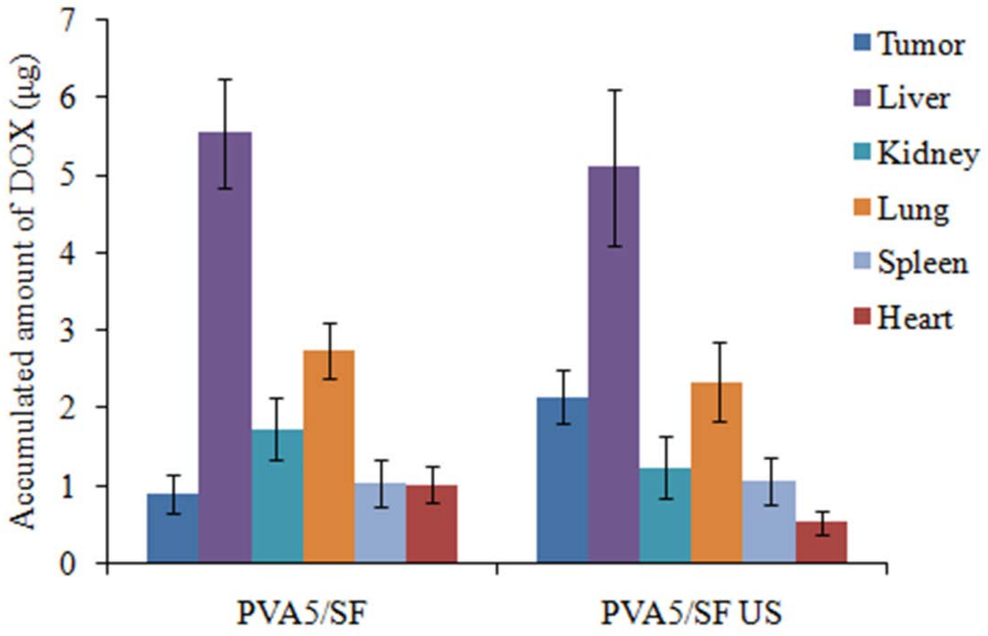

Figure S1. Accumulated DOX amount in vivo distribution. The released DOX was accumulated into the main reticuloendothelial organs, such as liver. By ultrasound radiation, the drug released and tumor targeting efficiency were significantly improved.

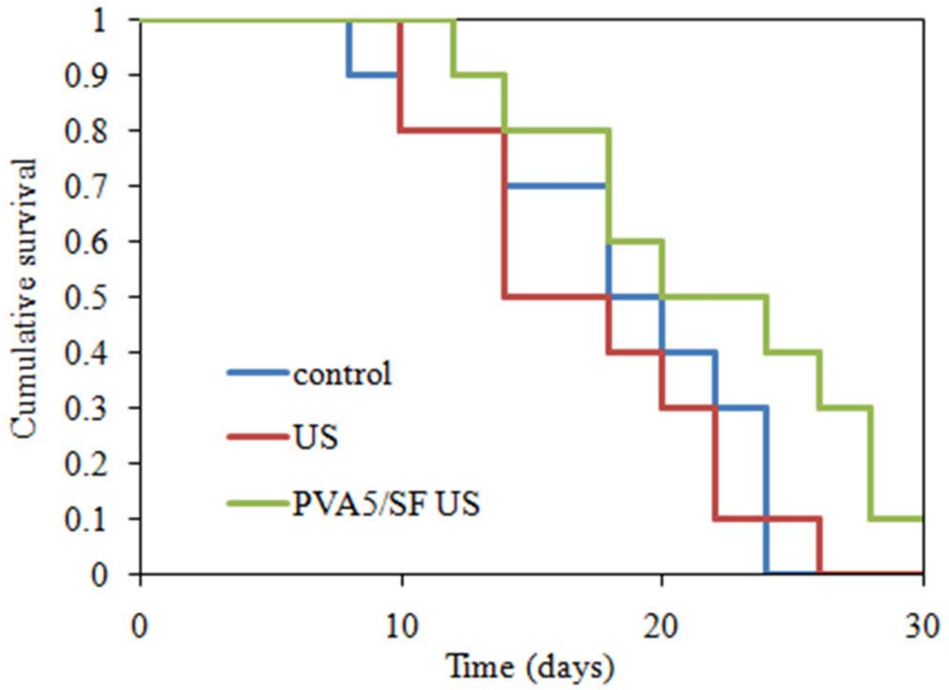

Figure S2. Cumulative survival of animals after intravenous administration of PVA5/SF nanoparticles and ultrasound irradiations.

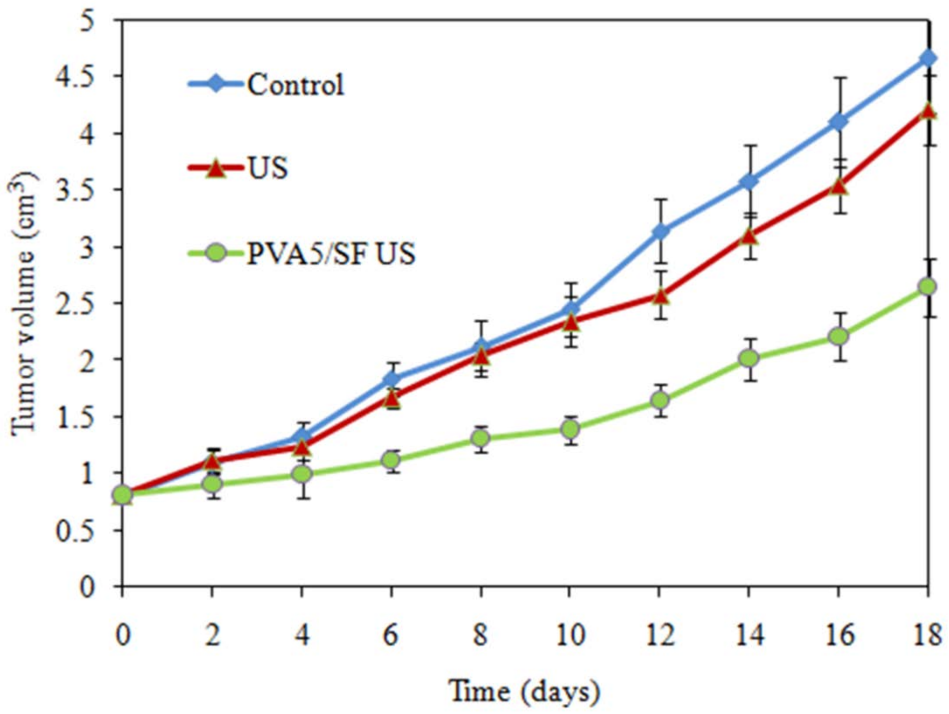

Figure S3. Tumor-volume curves after the injection of PVA5/SF nanoparticles followed by ultrasound treatment in vivo.
